# Supplementary material for: Early Childhood Junk Food Consumption, Severe Dental Caries, and Undernutrition: A Mixed-Methods Study from Mumbai, India
Source: Int J Environ Res Public Health. 2020 Nov 20;17(22):8629. doi: 10.3390/ijerph17228629 (PMC7699964; doi:10.3390/ijerph17228629)
Supplement: Supplementary file 1 [file ijerph-17-08629-s001.pdf]

Supplemental Table 1. Bivariate Associations Between Junk Food Consumption, Deep Caries and Undernutrition.

	BAZ				HAZ				WAZ				HAZ or BAZ or WAZ			
	Child Age < 3		Child age >= 3		Child Age < 3		Child age >= 3		Child Age < 3		Child age >= 3		Child Age < 3		Child age >= 3	
	OR	95% CI	OR	95% CI	OR	95% CI	OR	95% CI	OR	95% CI	OR	95% CI	OR	95% CI	OR	95% CI
**Consumption of junk food and sugary drinks**																
**Salty junk food**																
2-3 times per day vs rarely	0.47	0.22-1.00	0.82	0.41-1.65	0.93	0.52-1.68	1.10	0.64-1.9	**0.50**	**0.26-0.95**	0.92	0.53-1.6	**0.48**	**0.26-0.89**	0.75	0.44-1.28
Once a day vs rarely	0.74	0.39-1.43	1.36	0.73-2.57	0.73	0.42-1.28	0.96	0.57-1.61	0.63	0.35-1.13	1.07	0.63-1.81	**0.49**	**0.27-0.88**	0.87	0.53-1.45
Weekly vs rarely	0.99	0.48-2.03	1.33	0.68-2.58	0.65	0.35-1.24	1.35	0.79-2.34	0.53	0.27-1.06	1.51	0.87-2.6	0.56	0.29-1.09	1.17	0.68-2.00
**Sweet snacks**																
2-3 times per day vs rarely	**0.35**	**0.16-0.76**	0.73	0.37-1.46	1.48	0.83-2.63	0.60	0.35-1.03	0.82	0.44-1.52	0.75	0.43-1.28	0.83	0.46-1.50	**0.50**	**0.29-0.84**
Once a day vs rarely	0.50	0.24-1.05	1.40	0.71-2.75	1.44	0.79-2.62	0.76	0.44-1.33	0.59	0.3-1.17	1.24	0.71-2.17	0.67	0.36-1.23	0.83	0.47-1.45
Weekly vs rarely	0.65	0.34-1.25	1.41	0.73-2.74	1.05	0.59-1.85	1.05	0.61-1.8	0.88	0.48-1.6	1.20	0.7-2.09	0.77	0.43-1.37	0.95	0.55-1.63
**Sugar-sweetened beverages**																
2-3 times per day vs rarely	110	0.22-5.58	0.93	0.34-2.54	0.17	0.02-1.41	0.84	0.36-1.95	0.32	0.04-2.64	0.99	0.44-2.27	0.21	0.04-1.07	0.74	0.33-1.67
Once a day vs rarely	0.66	0.14-3.09	0.58	0.22-1.53	1.68	0.52-5.42	0.63	0.3-1.31	3.13	0.97-10.14	0.63	0.3-1.31	1.27	0.37-4.33	0.68	0.35-1.34
Weekly vs rarely	0.54	0.22-1.32	1.06	0.66-1.70	1.38	0.72-2.63	0.88	0.59-1.31	1.08	0.54-2.15	0.82	0.55-1.24	0.97	0.51-1.88	0.82	0.55-1.21
**Sugary tea**																
2-3 times per day vs rarely	0.42	0.02-1.12	1.00	0.47-2.11	1.47	0.58-3.72	0.99	0.53-1.86	1.58	0.59-4.21	0.88	0.44-1.75	0.87	0.34-2.21	1.15	0.64-2.07
Once a day vs rarely	0.68	0.32-1.42	**1.82**	**1.04-3.19**	1.70	0.93-3.11	1.55	0.96-2.52	1.17	0.6-2.30	**2.75**	**1.65-4.57**	1.28	0.69-2.4	**2.41**	**1.5-3.86**
Weekly vs rarely	1.00	0.30-3.31	0.72	0.23-2.30	2.21	0.75-6.48	1.17	0.49-2.8	0.73	0.2-2.72	**2.60**	**1.1-6.13**	1.96	0.6-6.4	1.38	0.6-3.17
**Severe dental caries**																
**Deep Caries**																
Has deep caries vs does not	1.03	0.21-5.06	1.43	0.94-2.18	0.14	0.02-1.14	0.72	0.50-1.04	NA*	NA*	1.18	0.82-1.70	1.03	0.21-5.06	0.32	0.08-1.31
**Deep Caries and Mouth pain**																
Has deep caries and mouth pain vs does not	**1.20**	**0.12-11.72**	1.44	0.90-2.33	NA*	NA*	0.74	0.48-1.14	NA*	NA*	1.23	0.81-1.86	0.22	0.02-2.11	1.06	0.70-1.60

*Sample size too small to estimate association
